# Effects of zinc supplementation and zinc chelation on in vitro β-cell function in INS-1E cells

**DOI:** 10.1186/1756-0500-7-84

**Published:** 2014-02-07

**Authors:** Sanne Bjørn Nygaard, Agnete Larsen, Astrid Knuhtsen, Jørgen Rungby, Kamille Smidt

**Affiliations:** 1 Department of Biomedicine, Centre of Pharmacology and Pharmacotherapy, Health, Aarhus University, Wilhelm Meyers Allé 4, Bld 1240, Aarhus, 8000, Denmark

**Keywords:** Zinc, Insulin, Zinc transporter, Metallothionein, Chelation, TPEN, INS-1E cells, β –cell, Diabetes

## Abstract

**Background:**

Zinc is essential for the activities of pancreatic β-cells, especially insulin storage and secretion. Insulin secretion leads to co-release of zinc which contributes to the paracrine communication in the pancreatic islets. Zinc-transporting proteins (zinc-regulated transporter, iron-regulated transporter-like proteins [ZIPs] and zinc transporters [ZnTs]) and metal-buffering proteins (metallothioneins, MTs) tightly regulate intracellular zinc homeostasis. The present study investigated how modulation of cellular zinc availability affects β-cell function using INS-1E cells.

**Results:**

Using INS-1E cells, we found that zinc supplementation and zinc chelation had significant effects on insulin content and insulin secretion. Supplemental zinc within the physiological concentration range induced insulin secretion. Insulin content was reduced by zinc chelation with *N*,*N*,*N*’,*N*-tektrakis(2-pyridylmethyl)-ethylenediamine. The changes in intracellular insulin content following exposure to various concentrations of zinc were reflected by changes in the expression patterns of MT-1A, ZnT-8, ZnT-5, and ZnT-3. Furthermore, high zinc concentrations induced cell necrosis while zinc chelation induced apoptosis. Finally, cell proliferation was sensitive to changes in zinc the concentration.

**Conclusion:**

These results indicate that the β-cell-like function and survival of INS-1E cells are dependent on the surrounding zinc concentrations. Our results suggest that regulation of zinc homeostasis could represent a pharmacological target.

## Background

Pancreatic tissue has high zinc (Zn^2+^) concentrations relative to other tissues because zinc is essential for its exocrine and endocrine functions [1]. In particular, Zn^2+^ is needed for the correct storage of insulin in secretory vesicles by ensuring that insulin forms crystalline structures [2]. Furthermore, Zn^2+^ is co-secreted with insulin and is involved in paracrine and autocrine communication within the pancreas [3]. Finally, Zn^2+^ regulates the activity of ATP-sensitive potassium (K_ATP_) channels and calcium (Ca^2+^) channels, which are involved in glucose-induced insulin secretion [4,5].

Abnormal zinc homeostasis seems to play an important role in impaired insulin sensitivity and diabetes. Diabetic subjects often display hypozincemia and hyperzincuria [6,7], and zinc deficient rats exhibit reduced insulin secretion and glucose sensitivity [8]. A local increase in Zn^2+^ concentrations cause pancreatic cell death by inducing apoptosis [9], while reductions in free zinc are associated with decreased insulin content in β-cells [10,11].

Cellular zinc homeostasis is tightly regulated because of the regulatory roles of intracellular Zn^2+^. Specialized proteins are responsible for controlling zinc import and export, as well as its intracellular distribution. Two classes of metal carrier proteins control the transmembrane transport of zinc ions. Zinc-regulated transporters and iron-regulated transporter-like proteins (ZIPs) facilitate Zn^2+^ influx into the cell and zinc transporters (ZnTs) facilitate Zn^2+^ efflux out of the cell [12,13]. The free zinc concentration is also influenced by the buffering activities of metallothioneins (MTs). MTs are a family of metal-binding proteins that are thought to maintain a reservoir of Zn^2+^ for use in cellular activities while simultaneously protecting against zinc toxicity [14,15]. Paradoxically, zinc supplementation and zinc depletion can be cytotoxic [16-20].

Modifying intracellular Zn^2+^ traffic by changing the gene expression levels of specific ZnT genes also affects β-cell insulin content and secretion [11,21-23]. The gene transcription of ZnTs and MTs is thought to be regulated by the intracellular zinc concentration, as demonstrated by studies using pancreatic islets, other cell lines (e.g., Caco-2 and HT-29 cells), and in some subsets of leukocytes [11,16-20,24,25]. Polymorphisms in the ZnT-8 gene are associated with glucose intolerance and type 2 diabetes [26-29]. Furthermore, streptozotocin (STZ)-treated ZnT-3–knockout mice exhibit impaired glucose metabolism compared with STZ-treated wild-type mice [11]. Overexpression of MTs was reported to prevent STZ-induced islet disruption, delay the onset of hyperglycemia in STZ-treated mice, and improve islet β-cell survival [30-32]. Finally, polymorphisms in genes encoding different isoforms of MTs were reported to be associated with the development of type 2 diabetes and diabetic complications [33,34].

Despite intensive research, the full consequence of altered zinc bioavailability on β-cell function remains unclear. Therefore, the present study investigated how cell survival, insulin content/secretion, and the expression of specific β-cell-relevant ZnTs and MTs respond to changes in the zinc environment following supplementation or chelation of zinc. We found that zinc-specific interventions had significant effects on the β-cell-like activity of INS-E1 cells, demonstrating the pharmacological potential of zinc supplementation or chelation.

## Methods

### Cell culture

Rat INS-1E cells were used in this *in vitro* study. The INS-1E cell line is an established glucose-sensitive cell line with β-cell-like activity [35,36]. INS-1E cells were cultured in a CO_2_ atmosphere in complete RPMI 1640 supplemented with 11 mM glucose, 10% (*v/v*) heat-inactivated fetal bovine serum, 50 μM β-mercaptoethanol, 2 mM L-glutamine, 100 U/ml penicillin, and 100 μg/ml streptomycin, as previously described [10,11,21]. The zinc concentration of this medium was approximately 2.5 μmol/l [10].

### Zinc supplementation and chelation

For stimulation assays, cells were plated into six-well plates (NUNC) in complete RPMI 1640 supplemented with 11 mM glucose, 10% (v/v) heat-inactivated fetal bovine serum, 50 μM β-mercaptoethanol, 2 mM L-glutamine, 100 U/ml penicillin, and 100 μg/ml streptomycin with the addition of either 5 μM to 1 mM zinc chloride (ZnCl_2_) (Merck, Germany) or 2.5–50 μM of the Zn^2+^ chelator *N*,*N*,*N*’,*N*-Tektrakis(2-pyridylmethyl)-ethylenediamine (TPEN) (Sigma Aldrich, Denmark). The basal glucose concentration was kept permanently at 11 mM because we experienced greater insulin response and cell replication of the INS-1E, and continuous growth at this concentration (unpublished data). We used 3–6 replicates for the analyses of mRNA expression, viability, DNA fragmentation assessment, and insulin measurements.

### Cell viability, cell cycle, and DNA fragmentation assay

INS-1E cells were treated with 50 μM to 1 mM ZnCl_2_ or 2.5–50 μM TPEN in complete RPMI medium for 24 h. Cells were harvested by trypsinization and samples pooled with cells floating in the used cell culture medium. The cells were partly collected in RPMI medium for assessing viability and partly in PBS for cell cycle and DNA fragmentation assays. Before analyzing cell cycle status and DNA fragmentation, the cells were transferred to ice-cold 70% ethanol, vortexed, and permeabilized at 0–4°C for ≥12 h. Cell cycle and DNA fragmentation were determined by incubating permeabilized cells in 1 μg/ml 4′,6-diamidino-2-phenylindole (DAPI) (Chemometec, Denmark), a DNA-specific dye, for 15 min at 37°C followed by fluorescence analysis on a NucleoCounter NC-3000 system (Chemometec). Viability was determined by analyzing cell samples on Via1-Cassettes (Chemometec) coated with two different dyes to stain the entire cell population (acridine orange) and nonviable cells (DAPI).

### Insulin assay

INS-1E cells were treated with 5 μM to 1 mM ZnCl_2_ or 2.5–50 μM TPEN in complete RPMI medium for 24 h. The cells were then incubated for 2 h in serum-free Krebs–Ringer bicarbonate HEPES buffer at pH 7.4 containing 115 mM NaCl, 4.7 mM KCl, 1.2 mM MgSO_4_, 2.6 mM CaCl_2_, 1.2 mM KH_2_PO_4_, 20 mM HEPES, 5 mM NaHCO_3_, 0.1% (*v/v*) human serum albumin (Sigma), with or without 50 μM to 1 mM ZnCl_2_ or 2.5–50 μM TPEN and 11 mM glucose. The incubation medium was collected to measure insulin secretion. The cells were collected in Earle’s basal medium (Invitrogen, Denmark) by scraping followed by centrifugation. Half of the intact cells from each sample were re-suspended in a buffer comprising 0.75% (*v/v*) glycine and 0.25% (*v/v*) bovine serum albumin at pH 8.8 to measure the insulin concentration, or in 0.1% M NaOH to measure the protein concentration. The total protein concentration was measured using a BCA Protein Assay Reagent Kit from Pierce, USA (Bie & Berntsen A/S, Denmark). The insulin concentration was determined using an ultrasensitive rat insulin enzyme-linked immunosorbent assay kit from DRG Diagnostics (VWR, Denmark).

### RNA extraction and cDNA synthesis

INS-1E cells were treated with 5 μM to 1 mM ZnCl_2_ or 2.5–50 μM TPEN in complete RPMI medium for 24 h. It was not possible to collect RNA material from cells treated with 50 μM TPEN most likely due to severe toxicity of TPEN at this concentration level. RNA was extracted using the RNeasy Mini Kit Qiagen (VWR) and treated with DNase (VWR). cDNA was synthesized from total RNA using an ImProm-II™ Reverse transcription system (Promega, Denmark).

### Real-time PCR

Quantitative real-time PCR was performed in duplicate using IQ Sybr Green supermix (Bio-Rad, Denmark) in a MyiQ Two-Color Real-time PCR detection system (Bio-Rad). A melting curve was prepared for all reactions. The results were analyzed with iQ^TM^5 Optical System Software, Version 2.1. Starting quantities were calculated from a standard curve. For each experiment, the most stable housekeeping genes were found using the method described by Vandesompele et al. [37]. Expression levels were normalized to the three most stable housekeeping genes from the following: β-actin, cyclophilin-A (CycA), heat shock protein (HSP), clathrin (Cltc), and ubiquitin-conjugase-7 (UBC-7). We selected the most stable housekeeping genes and normalized the data using previously reported methods [37,38].

### Primers used for real-time PCR

The following (forward and reverse) primers were used: UBC-7, 5′-CAG CTG GCA GAA CTC AAC AA-3′ and 5′-TTT GGG TGC CAA ATC TCT GT-3′ (annealing temperature 58°C); Cltc, 5′-AAG GAG GCG AAA CTC ACA GA-3′ and 5′-GAG CAG TCA ACA TCC AGC AA-3′ (annealing temperature 59°C); HSP, 5′-GAT TGA CAT CAT CCC CAA CC-3′ and 5′-CTG CTC ATC ATC GTT GTG CT-3′ (annealing temperature 59°C); CycA, 5′-AGG TCC TGG CAT CTT GTC CA-3′ and 5′-CTT GCT GGT CTT GCC ATT CC-3′ (annealing temperature 58°C); β-actin, 5′-CTA CAA TGA GCT GCG TGT GGC 3′ and 5′-ATC CAG ACG CAG GAT GGC ATG-3′ (annealing temperature 62°C); Bax, 5′-GTG AGC GGC TGC TTG TCT-3′ and 5′-GTG GGG GTC CCG AAG TAG-3′ (annealing temperature 59°C); Bcl-2, 5′-CGA CTT TGC AGA GAT GTC CA-3′ and 5′-ATG CCG GTC AGG TAC TCA G-3′ (annealing temperature 57°C); insulin, 5′-CGC TTC CTG CCC CTG CTG GC-3′ and 5′-CGG GCC TCC ACC CAG CTG CTC CA-3′ (annealing temperature 67°C); ZnT-3, 5′-TCC TCT TCT CTA TCT GCG CCC-3′ and 5′-TGT GCG GAG GCA ACG TGG TAA-3′ (annealing temperature 59°C); ZnT-5, 5′-TCC ACA TGC TCT TTG ACT GC-3′ and 5′-GTC AAG TTC CGG AGG ATC AA-3′ (annealing temperature 64°C); ZnT-8, 5′-GGT GGA CAT GTT GCT GGG AG-3′ and 5′-CAC CAG TCA CCA CCC AGA TG-3′ (annealing temperature 56°C); MT-1A, 5′-TCC CGA CTT CAG CAG CCC GA-3′ and 5′-GCC CTG GGC ACA TTT GGA GC-3′ (annealing temperature 63°C); and MT-3, 5′-TGG TTC CTG CAC CTG CTC GG-3′ and 5′-CAC CAG GGA CAC GCA GCA CT-3′ (annealing temperature 63°C).

### Statistical analysis

Data are presented as mean values with the standard error of the mean (SEM). One-way analysis of variance with Dunnett’s multiple comparison test was used to determine statistical significance among groups. Values of *P* < 0.05 were considered to indicate a significant difference between the experimental and control conditions.

## Results

### High zinc concentrations reduce INS-1E cell viability

**Figure 1:**
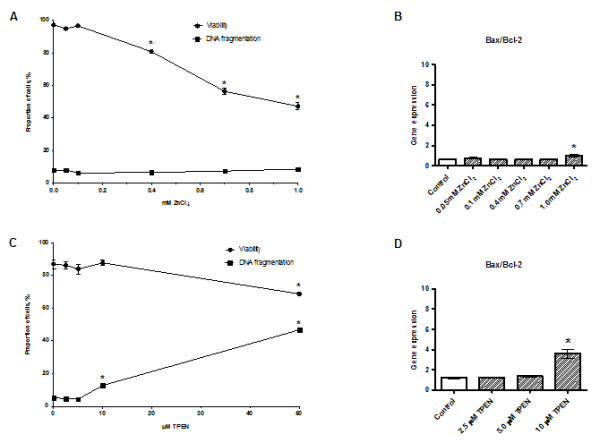
**Cell survival.** INS-1E cells were exposed to ZnCl_2_**(A, B)** or TPEN **(C, D)** for 24 h in the presence of 11 mM glucose. **(A, C)** cell viability and DNA fragmentation. **(B, D)** Bax/Bcl-2 gene expression. In cells exposed to ZnCl_2_, gene expression was normalized for β-actin, HSP, and Cltc. In cells exposed to TPEN, gene expression was normalized for HSP, CycA, and UBC-7. Data are shown as the mean ± SEM (*n* = 4–6). *P < 0.05.

The number of viable INS-1E cells decreased significantly when the ZnCl_2_ concentration reached 0.4 mM. The percentage of viable cells was decreased by 16.9% at 0.4 mM ZnCl_2_ and only 47.1% of the cells were viable at the highest ZnCl_2_ concentration, 1.0 mM (Figure 1A). Based on DNA fragmentation assays, treatment with ZnCl_2_ did not promote apoptosis (Figure 1A) and only a small increase in the Bax/Bcl-2 ratio was observed at 1.0 mM ZnCl_2_ (Figure 1B).

### Zinc chelation impairs INS-1E cell viability by inducing apoptosis

The viability of INS-1E cells decreased significantly by 18.2% following exposure to 50 μM TPEN (Figure 1C). DNA fragmentation was detected at 10 μM TPEN. Severe DNA fragmentation was observed at 50 μM TPEN and 41.4% of the cells exhibited reduced DNA content as a consequence of DNA fragmentation (Figure 1C). The Bax/Bcl-2 ratio was significantly increased in cells exposed to 10 μM TPEN (Figure 1D).

### The INS-1E cell cycle is affected by zinc supplementation

**Figure 2:**
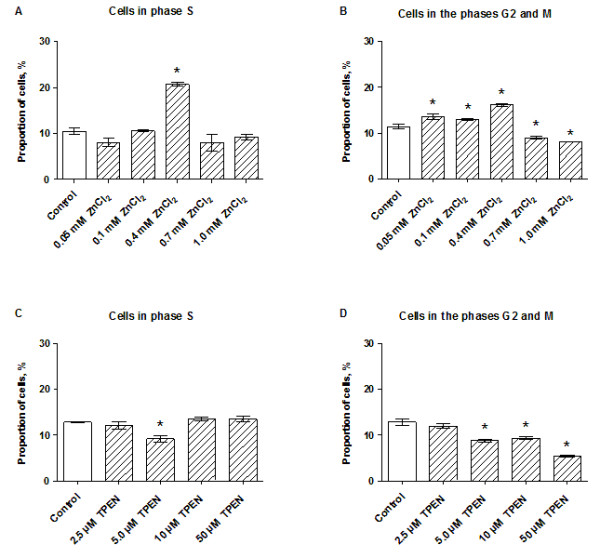
**Cell cycle.** The proportions of INS-1E cells in the S and G2/M phases were determined after exposure to ZnCl_2_**(A, B)** or TPEN **(C, D)** for 24 h in the presence of 11 mM glucose. Data are shown as the mean ± SEM (n = 4–6). *P < 0.05.

Supplementation with ZnCl_2_ disturbed the baseline distribution of cells in the different stages of the cell cycle (Figure 2A, B). Low ZnCl_2_ concentrations (0.05–0.4 mM) increased the proportion of cells in the G2/M phase while higher ZnCl_2_ concentrations (0.7–1.0 mM) reduced the number of cells in the G2/M phase. The fraction of cells in the S phase was also affected by the ZnCl_2_ concentration. The effect was particularly evident at 0.4 mM ZnCl_2_, where a two-fold increase in the number of cells was detected compared with the control cells (Figure 2A).

### Chelation of Zn^2+^ by TPEN reduces the proportion of dividing cells

The ratio of cells in the S phase was unaffected at all conditions tested, except in cells treated with 5.0 μM TPEN, where the proportion of cells was significantly decreased (Figure 2C). TPEN at concentrations ≥5.0 μM reduced the proportion of actively dividing cells in the G2/M phase (Figure 2C).

### Zinc is required to maintain baseline insulin secretion

**Figure 3:**
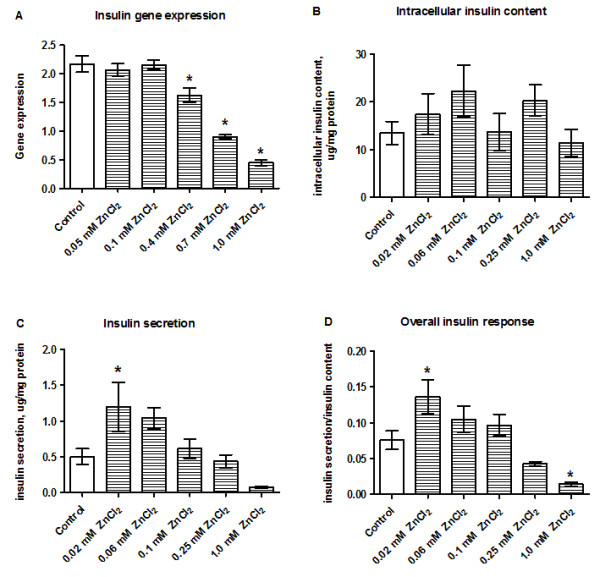
**Effects of zinc supplementation on insulin gene expression, insulin content and insulin secretion.** Insulin gene expression **(A)**, intracellular insulin content **(B)**, insulin secretion **(C)**, and the insulin secretion/content ratio **(D)** were assessed after INS-1E cells were stimulated with 20 μM to 1 mM ZnCl_2_ for 24 h in the presence of 11 mM glucose. Gene expression was normalized for β-actin, HSP, and Cltc. Data are shown as the mean ± SEM (*n* = 4–6). *P < 0.05.

**Figure 4:**
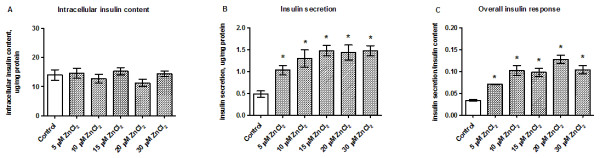
**Effects of physiological concentrations of ZnCl**_**2 **_**on insulin content and insulin secretion.** Intracellular insulin content **(A)**, insulin secretion **(B)**, and the insulin secretion/content ratio **(C)** were assessed after INS-1E cells were stimulated with 5–30 μM ZnCl_2_ for 24 h in the presence of 11 mM glucose. Data are shown as the mean ± SEM (*n* = 4). *P < 0.05.

Insulin gene expression was significantly reduced following exposure to cytotoxic concentrations of ZnCl_2_ (0.4–1.0 mM; Figure 3A). Although insulin content was unaffected by ZnCl_2_ (Figure 3B), the amount of secreted insulin was increased (Figure 3C), resulting in a significant increase in zinc-induced insulin secretion/insulin content ratio (Figure 3D). In an additional experiment using physiological concentrations of zinc (5–30 μM) we found no changes in the intracellular insulin content (Figure 4A). Insulin secretion increased in a dose-dependent manner across the concentration range of 5–10 μM ZnCl_2_ relative to the control group, and a plateau was reached at 15–30 μM ZnCl_2_ (Figure 4B). The insulin secretion/insulin content ratio at 5–15 μM ZnCl_2_ showed a similar pattern to the insulin secretion data (Figure 4C).

### Chelation of zinc by TPEN decreases the intracellular insulin content in INS-1E cells

**Figure 5:**
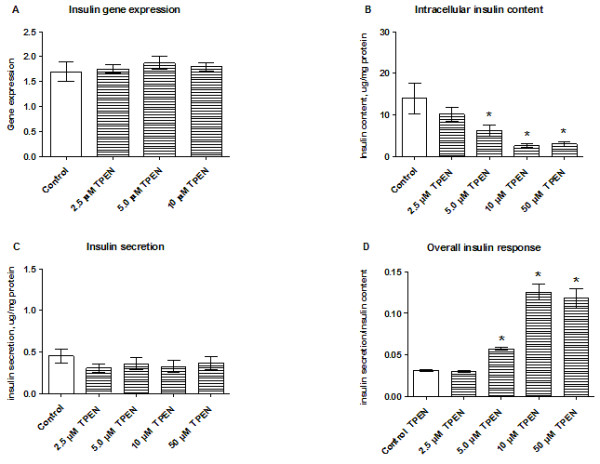
**Effects of zinc chelation on insulin gene expression, insulin content and insulin secretion.** Insulin gene expression **(A)**, intracellular insulin content **(B)**, insulin secretion **(C)**, and the insulin secretion/content ratio **(D)** were assessed after INS-1E cells were stimulated with 2.5–50 μM TPEN for 24 h in the presence of 11 mM glucose. Gene expression was normalized for HSP, CycA, and UBC-7. Data are shown as the mean ± SEM (*n* = 3–6). *P < 0.05.

Zinc chelation with TPEN did not affect insulin gene expression (Figure 5A). However, the intracellular insulin content was significantly reduced following exposure to 5.0, 10, or 50 μM TPEN (Figure 5B). Zinc chelation did not affect insulin release (Figure 5C), resulting in an increase in the overall insulin secretion/insulin content ratio (Figure 5D).

### ZnT-3 gene expression is markedly upregulated by zinc supplementation

**Figure 6:**
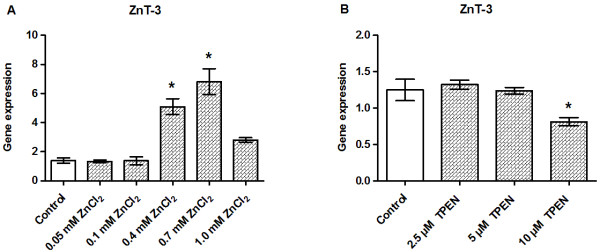
**Effects of zinc supplementation and zinc chelation on the gene expression levels of ZnT-3.** INS-1E cells were exposed to the indicated concentrations of ZnCl_2_**(A)** or TPEN **(B)** for 24 h in the presence of 11 mM glucose. The gene expression levels of ZnT-3 were normalized for β-actin, HSP, and Cltc in cells exposed to ZnCl_2_ and to HSP, CycA, and UBC-7 in cells exposed to TPEN. Data are shown as the mean ± SEM (*n* = 4–6). *P < 0.05.

ZnCl_2_ treatment significantly upregulated ZnT-3 transcriptions by 2–4.8-fold at concentrations ≥0.4 mM (Figure 6A). By contrast, Zn^2+^ chelation with 10 μM TPEN downregulated ZnT-3 gene expression (Figure 6B).

### ZnT-5 gene expression is downregulated by zinc chelation

**Figure 7:**
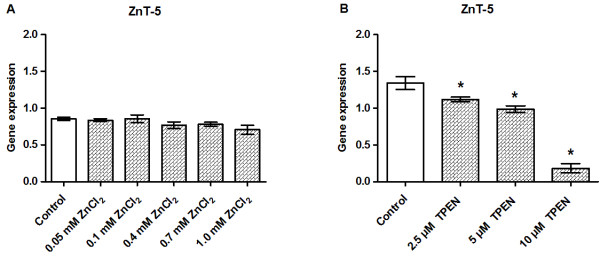
**Effects of zinc supplementation and zinc chelation on the gene expression levels of ZnT-5.** INS-1E cells were exposed to the indicated concentrations of ZnCl_2_**(A)** or TPEN **(B)** for 24 h in the presence of 11 mM glucose. The gene expression levels of ZnT-5 were normalized for β-actin, HSP, and Cltc in cells exposed to ZnCl_2_ and to HSP, CycA, and UBC-7 in cells exposed to TPEN. Data are shown as the mean ± SEM (*n* = 4–6). *P < 0.05.

ZnT-5 gene expression was not affected by zinc supplementation (Figure 7A) whereas chelation at high doses (5.0 and 10 μM) of TPEN resulted in downregulation of ZnT-5 gene expression (Figure 7B).

### ZnT-8 gene expression is sensitive to zinc supplementation and zinc chelation

**Figure 8:**
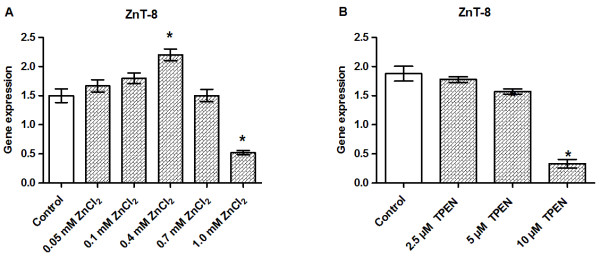
**Effects of zinc supplementation and zinc chelation on the gene expression levels of ZnT-8.** INS-1E cells were exposed to the indicated concentrations of ZnCl_2_**(A)** or TPEN **(B)** for 24 h in the presence of 11 mM glucose. The gene expression levels of ZnT-8 were normalized for β-actin, HSP, and Cltc in cells exposed to ZnCl_2_ and to HSP, CycA, and UBC-7 in cells exposed to TPEN. Data are shown as the mean ± SEM (*n* = 4–6). *P < 0.05.

ZnT-8 gene expression was gradually induced by zinc supplementation reaching statistical significance at 0.4 mM ZnCl_2_. The most cytotoxic ZnCl_2_ concentrations (0.7–1.0 mM) markedly reduced the transcription of ZnT-8 (Figure 8A). ZnT-8 gene expression was significantly downregulated by chelation with 10 μM TPEN (Figure 8B).

### MT-1A gene expression is upregulated by zinc supplementation without changes in MT-3

**Figure 9:**
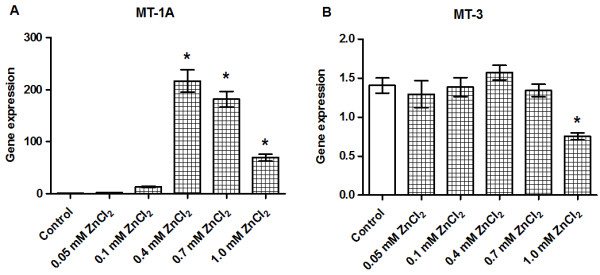
**Effects of zinc supplementation on the gene expression levels of metallothioneins.** INS-1E cells were exposed to the indicated concentrations of ZnCl_2_ for 24 h in the presence of 11 mM glucose. The gene expression levels of MT-1A **(A)** and MT-3 **(B)** were normalized for β-actin, HSP, and Cltc. Data are shown as the mean ± SEM (*n* = 4–6). *P < 0.05.

The gene expression of MT-1A was exceptionally sensitive to Zn^2+^ supplementation resulting in a transcriptional upregulation, 100 to 300-fold, at concentrations above 0.4 mM ZnCl_2_ (Figure 9A). By contrast, MT-3 transcription was only affected and was downregulated at the highest cytotoxic ZnCl_2_ concentration (1.0 mM ZnCl_2_; Figure 9B).

### Zinc chelation by TPEN downregulates MT-1A gene expression

**Figure 10:**
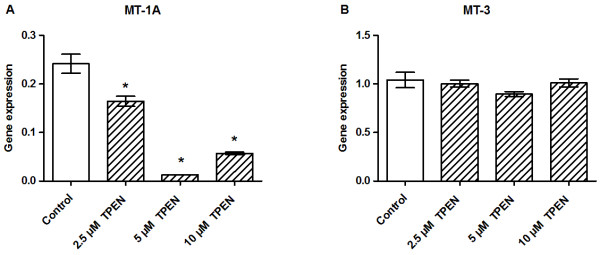
**Effects of zinc chelation on the gene expression levels of metallothioneins.** INS-1E cells were exposed to the indicated concentrations of ZnCl_2_ for 24 h in the presence of 11 mM glucose. The gene expression levels of MT-1A **(A)** and MT-3 **(B)** were normalized for HSP, CycA and UBC-7. Data are shown as the mean ± SEM (*n* = 4–6). *P < 0.05.

Zn^2+^ chelation with TPEN significantly downregulated MT-1A gene expression (Figure 10A) but did not affect MT-3 expression (Figure 10B).

## Discussion

Using INS-1E cells, this study demonstrated that manipulation of the zinc environment may affect β-cell survival and insulin production by interfering with intracellular zinc homeostasis under the control of the zinc transporters ZnT-3, ZnT-5, and ZnT-8. Excess zinc supply seems to reduce the viability of INS-1E by causing cellular necrosis. Synaptic Zn^2+^ release was reported to be related to exocytotic neuronal death [39,40]. In this mechanism, zinc was reported to cause cell death by re-entering neurons through ZnTs, *N*-methyl-D-aspartate receptor-mediator channels, and voltage-dependent calcium channels. Here, we find that zinc at concentrations of up to 0.1 mM ZnCl_2_ is well tolerated by INS-1E cells, but increasing the concentration from 0.2 to 1 mM steadily increased cell death. At 1 mM ZnCl_2_, 52.8% of INS-1E cells were dead. The concentration of Zn^2+^ within the insulin granules is approximately 20 mM [40,41] and, upon glucose stimulation, the concentration of Zn^2+^ co-secreted into the extracellular space may reach 475 μM [9], corresponding to the concentration of 400 μM (0.4 mM) that significantly increased cell death in our study (Figure 1A). Our results indicate that an excessive extracellular Zn^2+^ load, resulting from insulin release, may promote β-cell death, which might be particularly important in the context of hyperinsulinemia. Reductions in Zn^2+^ packaging might also result in an increase in free labile zinc, increasing β-cell damage. A similar cytotoxic effect might occur in autoimmune diabetes because an increase in secretory granular Zn^2+^ release occurs alongside the loss of β-cells [42-44]. Several studies have suggested that limiting cellular Zn^2+^ concentrations by reducing dietary zinc uptake or administering a low-affinity Zn^2+^ chelator, such as clioquinol, could attenuate the development of the diabetic state resulting from zinc accumulation [43,44].

MT-1A is abundantly expressed and is generally considered to have a protective role against oxidative stress. MT-1A is essential for the regulation of intracellular zinc homeostasis because it acts as a Zn^2+^ acceptor and a Zn^2+^ donor to control the availability of cellular zinc [45]. MT-1A gene expression is controlled by metal response element-binding transcriptional factor (MTF)-1 [46], allowing free Zn^2+^ to directly control the transcription of MT-1A. Notably, in the present study, we found that MT-1A responded strongly to changes in the zinc concentration. MT-1A upregulation was pronounced following zinc supplementation. Similar results were also observed in pancreatic islets [18,24], indicating that excess extracellular zinc causes an increase in intracellular free Zn^2+^, a process that is possibly mediated by the ZnT-1 transporter.

MT-3 is predominately expressed in the brain, where it acts as a neuronal growth inhibition factor with neuroprotective properties [47]. Although MT-3 has been localized in peripheral tissues, its role in these tissues is not understood [14,48]. Unlike MT-1A, the expression of MT-3 does not seem to be controlled by MTF-1. Consistent with this, we found that changes in the environmental Zn^2+^ concentration did not directly affect MT-3. However, we did observe transcriptional downregulation of MT-3 after exposing cells to highly cytotoxic conditions, such as 1.0 mM ZnCl_2_, and we expect this to be caused by the ongoing processes underlying cell death in these conditions. The results of this study support those of other studies indicating that MT-3 plays a different role to MT-1A in the pancreas, and that MT-3 is unlikely to be a direct regulator of intracellular zinc signaling in β-cells.

In neurons, ZnT-3 transports zinc ions into synaptic vesicles. This Zn^2+^ transporter is also expressed in β-cells [11,14,49,50]. The increase in ZnT-3 gene expression observed in the present study is consistent with our previous finding [11] that ZnT-3 is upregulated during stressful conditions (Figure 6A).

Expression of ZnT-8 is highly tissue-specific and besides β-cells, ZnT-8 is also expressed in adipose tissue and in the retina [14,39,51]. ZnT-8 is thought to be crucial for β-cell function because it is thought to transport zinc ions into insulin-containing secretory vesicles [23,52]. Here, we found that ZnT-8 is upregulated by exposure to low, non-cytotoxic ZnCl_2_ concentrations, indicating that Zn^2+^ uptake into insulin-containing granules is increased if Zn^2+^ is readily available. This is supported by other findings showing that INS-1E cells overexpressing ZnT-8 have higher intracellular Zn^2+^ concentrations compared with wild-type cells [21]. It is possible that this regulatory mechanism has a protective role because ZnT-8 overexpression was reported to protect β-cells from zinc depletion because of enhanced storage capacity [22]. It seems that ZnT-8 gene expression is correlated with the cellular zinc content in β-cells, as observed in RPE cells [51]. At a functional level, the zinc supplementation study confirmed the importance of the constant presence of Zn^2+^ in controlling insulin secretion (Figure 4C). In this study, immediate insulin secretion was compared between a basal zinc environment and a Zn^2+^-supplemented environment. Overall, we found that zinc, at physiological concentrations [1,53,54] of 15–30 μM, increased the release of insulin from INS-1E cells, emphasizing the importance of Zn^2+^ as a regulator of glucose-induced insulin secretion under normal conditions. This effect of zinc supplementation was demonstrated in a previous study using pancreatic islets, in which it was proposed that Zn^2+^ is an autocrine signaling molecule in the endocrine pancreas [55].

The pivotal role of Zn^2+^ in the regulation of insulin secretion is also reflected by the chelation experiments using TPEN. TPEN preferentially chelates free Zn^2+^, but also depletes zinc ions that are tightly bound to cellular metallo-proteins when administered at high concentrations. The effect of zinc chelation by TPEN on insulin secretion has not been examined in prior studies. We found that the predominant effect of chelation in INS-1E cells involves a reduction in the intracellular insulin content. Because insulin crystallization is an essential function of Zn^2+^, a reduction in intracellular insulin could be a consequence of impaired insulin storage in conditions of inadequate zinc. These results are consistent with our previous studies showing that the insulin content is reduced in β-cells exposed to the chelator diethyldithiocarbamate (DEDTC) [11].

ZnT-8 gene expression was reported to be downregulated by the chelator DEDTC [11,38], although this effect was less pronounced in the present study. The effects of zinc chelation by TPEN and DEDTC have been investigated in the context of hippocampal excitability. In the hippocampus, TPEN and DEDTC had different effects, suggesting that the discrepancy is caused by the more specific Zn^2+^ binding by TPEN than DEDTC, and by the fact that DEDTC chelates other metals, including copper [56]. In addition, TPEN may favor Zn^2+^ because TPEN has a higher binding affinity for Zn^2+^ (dissociation constant 1–2.6 × 10^-16^ M) than for other metals [57].

TPEN-based Zn^2+^ chelation also reduced the viability of INS-1E cells. Administered *in vitro*, TPEN can chelate extracellular zinc ions, cytoplasmic-free Zn^2+^, zinc located within intracellular compartments, and strip Zn^2+^ from proteins [58]. Thus, exposing β-cells to TPEN is expected to reduce the availability of zinc and interfere with zinc-dependent cellular activities. DNA fragmentation assays showed that chelation is stressful to INS-1E cells, and initiates programmed cell death, even though Bax/Bcl-2 activity was unaffected after 24 h of stimulation, except at the most cytotoxic stimuli (Figure 1D). Monitoring Bax/Bcl-2 at an earlier time might have revealed an altered ratio. The pro-apoptotic effects of zinc deficiency were previously demonstrated in several other cell lines [16,22,25,59,60]. TPEN directly affected the distribution of cells in different stages of the cell cycle, reflecting the importance of Zn^2+^ in cell division. It seems likely that Zn^2+^ is required for the passage of cells through the cell cycle. Certainly, several DNA-synthesizing enzymes seem to depend on Zn^2+^, suggesting that zinc depletion suppresses DNA synthesis [61,62].

Unlike other zinc transporters, ZnT-5 holds a unique position in regulating intracellular Zn^2+^ concentrations because it is localized in the Golgi apparatus, secretory vesicles, and in the cell membrane [63,64]. ZnT-5 is also implicated in Zn^2+^ efflux and influx. A study of ZnT-5–knockout mice revealed a reduction in islet zinc content in these animals [43]. Although the protein is abundant in pancreatic tissue [65], little is known about the function of ZnT-5 in this organ. In our experiments, ZnT-5 gene expression was not substantially affected by zinc supplementation but was sensitive to chelation by TPEN. This downregulatory effect of TPEN on ZnT-5 gene expression differs from that of studies using other cell types. ZnT-5 was reported to be upregulated by TPEN in Hela epithelial cells [25] and was upregulated or unaffected by TPEN in some subtypes of leukocytes [17]. Thus, the role of ZnT-5 in cellular Zn^2+^ homeostasis may be tissue-specific and might be related to the role of free Zn^2+^ in individual cell types.

## Conclusion

Using INS-1E cells, the present results indicate that β-cell function is directly related to the surrounding Zn^2+^ concentration, adding to the accumulating evidence that links abnormal zinc homeostasis to the development of diabetes. Manipulation of the cellular zinc environment was found to have significant effects on cell survival, cell proliferation, and insulin processing and release. Understanding the finely tuned system involved in zinc transport and zinc buffering might open a new field of research into pharmacological intervention aimed at prolonging and improving pancreatic β-cell function.

## Competing interests

The authors declare that they have no competing interests.

## Authors’ contributions

SBN, AL, JR, and KS conceived and designed the experiments, and wrote the manuscript. SBN and AK carried out the experiments. SBN, AL, AK, JR, and KS analyzed the data and approved the final version to be published. All authors read and approved the final manuscript.
